# Blackcurrant (*Ribes nigrum* L.) Matrices: Polyphenol Release, Antioxidant Capacity and Enzyme Inhibitory Potential

**DOI:** 10.3390/antiox15070783

**Published:** 2026-06-23

**Authors:** Martyna Szydłowska, Aneta Wojdyło, Paulina Nowicka

**Affiliations:** Department of Fruit, Vegetable and Plant Nutraceutical Technology, Wrocław University of Environmental and Life Sciences, 37 Chełmońskiego St., 51-630 Wrocław, Poland; martyna.szydlowska@upwr.edu.pl (M.S.); aneta.wojdylo@upwr.edu.pl (A.W.)

**Keywords:** blackcurrant, seeds, leaves, bioactive compounds, in vitro digestion, antioxidant activity, pro-health properties by in vitro

## Abstract

Blackcurrant (*Ribes nigrum* L.) fruits and their by-products represent valuable sources of bioactive compounds. This study compared fruits, juice, seeds, oil, and leaves with respect to their content of selected bioactive components, potential intestinal availability of polyphenols estimated by dialysis, and in vitro biological activities. Blackcurrant leaves contained several-fold higher levels of polyphenols in the dialyzable fraction (651.3 mg/100 g) than fruits (255.1 mg/100 g) and juice (261.4 mg/100 g). Seeds exhibited the strongest antioxidant activity among all matrices, reaching 13.3, 10.9 and 11.4 mmol Trolox/100 g in the ABTS, FRAP and ORAC assays, respectively. Hydrophilic fractions of juice and seeds showed notably stronger α-amylase inhibition (IC_50_ < 0.01 mg/mL) than the antidiabetic drug acarbose (IC_50_ = 0.35 mg/mL). Juice also demonstrated higher pancreatic lipase inhibition (IC_50_ = 0.01 mg/mL) compared with Orlistat (IC_50_ = 0.15 mg/mL) and effectively inhibited acetylcholinesterase, butyrylcholinesterase, and 15-lipoxygenase (IC_50_ = 0.11, 0.03, and 0.02 mg/mL, respectively). These results indicate that various blackcurrant matrices possess strong biological activity and may serve as promising functional food ingredients or sources of health-promoting compounds.

## 1. Introduction

Berries are a well-known raw material valued by both consumers and the food industry. Their popularity stems from their flavor, smell, color, and chemical composition. Importantly, berries are recognized for their potential health-promoting effects, including antioxidant and anticancer activity. In particular, in vitro and in vivo studies have demonstrated that bioactive compounds present in berries may inhibit the proliferation of cancer cells and modulate mechanisms associated with cancer development, including liver, colon, breast, and prostate cancers [1]. Blackcurrant (*Ribes nigrum* L.) is a notable representative of berries. It is widely cultivated throughout Europe, with annual production exceeding 130,000 tons, of which approximately 70% is produced in Poland, making it the leading producer within Europe. The fruit is used both for direct consumption and, predominantly, for industrial processing into juices, concentrates, frozen products, and jams, reflecting its considerable economic and nutritional importance. In addition, their fruits are rich in polyphenolic compounds, dietary fiber, pectins, vitamins, and minerals [2,3]. Blackcurrants are particularly distinguished by their high concentration of vitamin C and organic acids, while their seeds contain polyunsaturated fatty acids. The major polyphenolic compounds found in blackcurrants include anthocyanins (predominantly delphinidin and cyanidin derivatives), flavonols, phenolic acids, and flavan-3-ols. In addition, the fruits are a valuable source of minerals, such as potassium, calcium, and magnesium, whereas the seeds are rich in unsaturated fatty acids, particularly linoleic and α-linolenic acids [1]. Literature sources indicate that blackcurrant fruits are highly beneficial, helping to prevent cardiovascular and certain eye diseases, and improving blood plasma lipid levels [1,2]. Studies have also highlighted the potential antioxidant, anti-inflammatory, and anticancer properties of blackcurrant fruits, though evidence from randomized controlled human trials remains limited [2].

However, while consumers and the industry focus on the benefits of fruits of various species and their processing possibilities, they pay less attention to other parts of the plants. Meanwhile, numerous reports indicate that other morphological parts of plants, such as leaves, seeds, skins, flowers, and stems, are also valuable sources of bioactive compounds [4,5,6]. Blackcurrant leaves are particularly rich in flavonols, phenolic acids, and flavan-3-ols [5], whereas the seeds contain substantial amounts of polyunsaturated fatty acids, tocopherols, and other lipophilic bioactive compounds [6]. These characteristics suggest that different morphological parts of blackcurrant may provide distinct biological activities and functional applications beyond those traditionally associated with the fruits. Therefore, research on these underutilized plant parts could benefit both the food and pharmaceutical industries by identifying new raw materials with potential applications. Consumers could experience improved health from consuming products enriched with these materials or compounds derived from them. Moreover, utilizing various plant parts could provide economic benefits and align with the principles of sustainable development.

Although blackcurrant fruits are widely recognized as valuable sources of bioactive compounds, other morphological parts of the plant, such as leaves and seeds, remain considerably less explored despite their potential nutritional and functional value. A comprehensive characterization of these materials may support their valorization and facilitate their use in food, nutraceutical, and pharmaceutical applications, thereby reducing the underutilization of plant resources and promoting a more sustainable use of blackcurrant biomass. Moreover, most previous studies have focused on individual blackcurrant matrices or selected groups of bioactive compounds, whereas comparative investigations integrating phytochemical composition, potential intestinal availability of polyphenols estimated by dialysis, and multiple biological activities across different blackcurrant-derived materials remain scarce.

This study provides a comprehensive overview of the biologically active compound profiles of fruits, juice, seeds, seed oil, and leaves of blackcurrants. It evaluates their in vitro health-promoting potential, including antioxidant activity and the ability to inhibit α-amylase, α-glucosidase, pancreatic lipase, acetylcholinesterase (AChE), butyrylcholinesterase (BuChE), and 15-lipoxygenase (LOX-15). The study also examines the in vitro potential bioaccessibility and intestinal availability of polyphenolic compounds estimated by dialysis in the tested fruits, juices, and leaves.

To the best of our knowledge, no previous study has simultaneously compared blackcurrant fruits, juice, seeds, seed oil, and leaves with respect to their phytochemical composition, potential polyphenol bioaccessibility, and such a broad spectrum of biological activities. Specifically, the potential bioaccessibility and intestinal availability of polyphenolic compounds estimated by dialysis in these materials has not been investigated. The ability of berries and their products to inhibit AChE and BuChE remains largely unexplored. Additionally, the activity of different morphological parts of blackcurrant in this regard, as well as their ability to inhibit pancreatic lipase and LOX-15, has not been examined. This study, therefore, provides a broader understanding of the fruits, juice, seeds, seed oil, and leaves of blackcurrants in the context of their technological, nutraceutical, and pharmaceutical potential.

## 2. Materials and Methods

**Figure 1:**
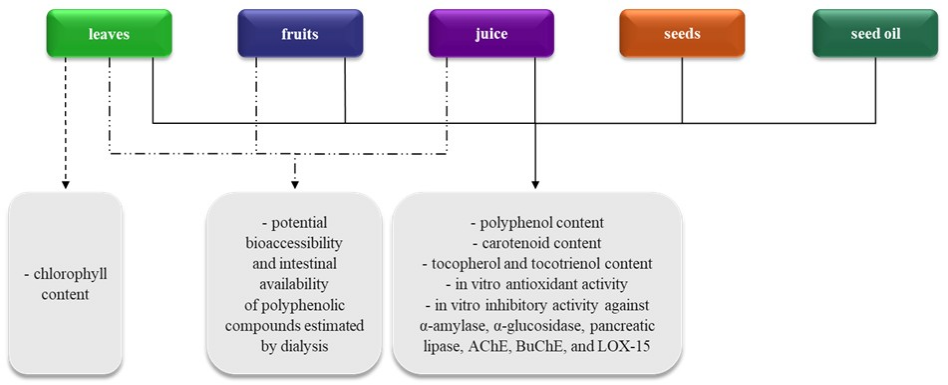
Schematic overview of the experimental design used in the study.

To facilitate understanding of the experimental design, a schematic overview of the study is presented in Figure 1. Blackcurrant fruits, juice, seeds, seed oil, and leaves were analyzed for their bioactive compound composition, while antioxidant and enzyme inhibitory activities were evaluated separately in hydrophilic and hydrophobic fractions. Simulated digestion and the assessment of potential polyphenol bioaccessibility and intestinal availability of polyphenolic compounds estimated by dialysis were performed for fruits, juice, and leaves.

### 2.1. Chemicals and Reagents

The reagents used to analyze in vitro pro-health activities and potential bioaccessibility, including acarbose, acetic acid, acetylthiocholine iodide, AChE, ascorbic acid, bile extract, butyrylcholine chloride, BuChE, dimethyl sulfoxide (DMSO), FeCl_3_, fluorescein disodium (FL), lipase, methanol, *p*-nitrophenyl-α-D-glucopyranoside, pancreatin, pepsin, phloroglucinol, starch, tacrine, 2,2′-azinobis-(3-ethylbenzthiazoline-6-sulfonic acid) (ABTS), 2,2′-azobis (2-amidino-propane) dihydrochloride (AAPH), 2,4,6-tripyridyl-1,3,5-triazine (TPTZ),6-hydroxy-2,5,7,8-tetramethylchroman-2-carboxylic acid (Trolox), α-amylase, and α-glucosidase were purchased from Sigma-Aldrich (Steinheim, Germany).

### 2.2. Sample Preparation

The fruits and leaves were collected in July 2022, when the fruits had reached full technological maturity. The blackcurrant variety “Tiesel” was collected from the village of Próba, near Sieradz. To obtain juices, the fruits were pressed using a laboratory hydraulic press (SRSE, Warsaw, Poland). To extract seeds, the fruits were crushed, rubbed through a sieve, and washed under running tap water. The seeds were then cleaned manually, placed on glass dishes, and left to dry completely. Immediately after collection, the fruits were freeze-dried and stored at 4 °C in the absence of light until analysis. The remaining fruits were freeze-dried, while the leaves were air-dried at room temperature for approximately 72 h and protected from direct light during drying. Once dried, the samples were ground using an electric grinder (TSM6A013B, Bosch, Nazarje, Slovenia). Cold-pressed blackcurrant seed oil without additives was produced by Olvita Gołuch sp.k. (Mysłaków, Poland). The results are presented on a fresh weight basis and were recalculated according to the dry matter content of the individual plant materials.

### 2.3. Content of Polyphenols, Including Polymers Procyanidins, by UPLC

The samples were extracted by mixing 0.5 g of sample with 5 mL of 30% MeOH containing ascorbic acid, and subjected to ultrasonication in an ultrasonic bath (*t* = 15 min). They were then stored in a refrigerator (*t* = 20 h) and ultrasonicated again (*t* = 15 min). Subsequently, the samples were centrifuged in a laboratory centrifuge (*t* = 10 min), and the supernatant was decanted and used for chromatographic analysis, performed following the methodology described by Nowicka et al. [7]. Polyphenols were analyzed using an ACQUITY UPLC system (Waters Corporation, Milford, MA, USA) equipped with an ACQUITY BEH C18 column (2.1 × 100 mm, 1.7 μm). The chromatographic separation was performed at a flow rate of 0.42 mL/min with a sample injection volume of 5 μL and a total run time of 15 min. The mobile phase consisted of 0.1% formic acid in water (solvent A) and acetonitrile (solvent B). A gradient elution program was applied, starting with 99% A and decreasing to 65% A over 12 min, followed by column conditioning and re-equilibration to the initial conditions. Polyphenolic compounds were detected at 280 nm (flavan-3-ols), 320 nm (phenolic acids), 360 nm (flavonols), and 520 nm (anthocyanins). Polyphenolic compounds were identified using an in-house library of authentic reference standards, including cyanidin-3-O-rutinoside, delphinidin-3-O-rutinoside, chlorogenic acid, quercetin derivatives, and (+)-catechin.

The determination of polymeric procyanidin content began with freeze-drying juice samples weighing approximately 0.500 g in Eppendorf tubes (Hamburg, Germany). For dried fruits, seeds, and leaves, approximately 50 mg of each sample was weighed into the same type of tube. To all dry samples, 0.8 mL of phloroglucinol and 0.4 mL of 2-methanol were added. The contents were mixed using a vortex mixer and then shaken using a BioSan Multi RS-60 rotator (Otwock, Poland) for 30 min (*T* = 50 °C, RPM = 800). The samples were cooled in a cooling chamber, and 0.6 mL of sodium acetate was added. The samples were then centrifuged (*t* = 5 min, RPM = 14,000).

After centrifugation, 0.6 mL of each sample was transferred to new tubes containing 0.6 mL of precooled ethyl acetate stored in a refrigerator, and then they were centrifuged in a laboratory centrifuge (*t* = 5 min, RPM = 14,000). The clear liquid was decanted into vials and analyzed according to the protocol described by Kennedy and Jones [8]. Results are presented as mg/100 g ± SD.

### 2.4. Potential Bioaccessibility and Intestinal Availability of Polyphenolic Compounds Estimated by Dialysis

A simulated digestion process was conducted to recreate the conditions of the digestive system, following the methodology described by Świeca et al. [9] with modifications. Previously lyophilized samples (lyophilized using an Alpha 1–4 LSC plus freeze dryer, Martin Christ, Osterode am Harz, Germany) were weighed to four decimal places: approximately 1 g for leaves and fruits, and about 20 g for juices. Sample weights were adjusted according to the dry matter content of the analyzed materials. Approximately 20 g of juice was required to obtain a dry matter content comparable to that present in 1 g of the freeze-dried samples. Simulated salivary fluid was added to the samples, and the mixtures were shaken (*t* = 10 min, *T* = 37 °C). The pH of the samples was then adjusted to 3 using 5 mol/L HCl, followed by the addition of simulated gastric fluid. These mixtures were shaken (*t* = 60 min, *T* = 37 °C). Subsequently, the pH of the samples was adjusted to 6 using 0.1 mol/L NaHCO_3_, and a mixture of bile extract and pancreatin was added. The pH was further adjusted to 7 using 1 mol/L NaOH. Finally, NaHCO_3_ (120 mmol/L) and KCl (5 mmol/L) were added to the samples. In vitro digestion was carried out in the dark for 120 min (*T* = 37 °C).

After digestion, 10 mL of digesta was transferred into cellulose dialysis sacks (D9777–100FT, Sigma-Aldrich, St. Louis, MO, USA; MWCO 12–14 kDa), and each sack was immersed in a separate Erlenmeyer flask containing 50 mL of PBS buffer, resulting in a sample-to-buffer ratio of 1:5 (*v*/*v*). Dialysis was carried out in a rotary shaker *(t* = 120 min, *T* = 37 °C) in two independent replicates. Compounds that passed through the membrane into the PBS buffer were collected as the dialyzable fraction and considered potentially available for intestinal absorption. The buffer-compound mixtures were collected, lyophilized, supplemented with a mixture of MeOH containing 2% formic acid, and mixed for 30 min using a rotator. The mixtures were centrifuged twice (*t* = 5 min, RPM = 14,000), with the supernatant transferred to empty tubes (Eppendorf, Hamburg, Germany) after each centrifugation. Chromatographic analysis of the polyphenolic compound content was then performed using previously described methodologies.

### 2.5. Content of Carotenoids, Tocopherols, and Tocotrienols

The analyzed materials included lyophilized fruits, juices, and leaves, dried seeds ground prior to extraction, and cold-pressed blackcurrant seed oil. Samples weighing 2–3 g were placed in falcons. Five milliliters of EtOH with BHT (0.05%) and 1 mL of 60% KOH were added to seeds and oil. In addition to the previously mentioned amounts of reagents, 5 mL of EtOH with BHT was added to fruits and leaves. In the case of juices, in addition to the reagents used for seeds and oil, 2 mL of EtOH was added. The samples were heated in a water bath (*t* = 1 h, *T* = 80 °C). After cooling, 10 mL of 1% NaCl and 10 mL of hexane:ethyl acetate (9:1) mixture were added to the samples and shaken (*t* = 40 min) using a rotator. Oil was an exception; due to its specific nature, it was shaken manually several times over 40 min.

For all mixtures except oil, 3 mL of a saturated NaCl solution was added. The upper layers, which separated in the tubes, were transferred to new tubes and evaporated to dryness using an XcelVap system (Horizon Technology, Inc.;Salem, NH, USA) under a gentle stream of nitrogen at 40 °C. Another 10 mL of the hexane:ethyl acetate (9:1) mixture was added to the samples, and the process was repeated twice more.

The final sediment collected after evaporation was treated with a mixture of tetrahydrofuran, methanol, and BHT: 2 mL for leaves and 1 mL for other samples. The samples were mixed, centrifuged at 15,000 rpm (10 min; 20 °C) using an MPW 352R laboratory centrifuge (MPW Med. Instruments; Warsow, Poland), and the supernatant was decanted. This centrifugation and decanting process was repeated, and the resulting liquid was transferred to analytical vials.

Carotenoid content analysis was conducted following the method described by Turkiewicz et al. [10]. Carotenoids were analyzed using an ACQUITY UPLC system (Waters Corporation, Milford, MA, USA) equipped with a RP C18 column (dimensions: 0.21 × 10 cm, 1.7 μm, Waters; USA). The injection volume was 10 μL, a gradient separation over 16.60 min was performed with a mixture of methyl cyanide:methyl alcohol (70%:30%) and 0.1% methanolic acid, the flow rate was 0.500 mL/min, and detection was performed at 430 and 450 nm. Results are presented as mg/100 g ± SD.

Chromatographic analysis of tocopherol and tocotrienol content was performed as described by Turkiewicz et al. [11]. Tocopherols and tocotrienols were analyzed using an ACQUITY UPLC system (Waters Corporation, Milford, MA, USA) equipped with a column (BEH Shield RP18, 130 Å, 1.7 µm, 2.1 × 100 mm, Waters Corp., Milford, MA, USA). The injection volume was 10 μL, the flow rate was 0.45 mL/min (isocratic elution with methanol and formic acid (0.1% *v*/*v*) in the proportion 88:12 (*v*/*v*)). The wavelengths were: excitation (λ = 290 nm), and emission (λ = 330 nm). Results are presented as mg/kg ± SD due to the relatively low concentrations of these compounds in the analyzed plant matrices.

### 2.6. Content of Chlorophylls

Approximately 200 mg of each sample was combined with 6 mL of hexane:acetone:methanol (2:1:1, *v*/*v*/*v*) reagent. The mixture was ultrasonicated for 60 min and then centrifuged in a laboratory centrifuge (*t* = 5 min, RPM = 10,000, *T* = 10 °C). The supernatant was transferred to test tubes and evaporated.

To the resulting precipitate, 2 mL of a MeOH:THF (1:1) mixture was added. The mixture was purified using syringe filters and transferred to analytical vials. The analysis was then performed following the method described by Turkiewicz et al. [10] using the same UPLC system and chromatographic conditions as those described for carotenoid analysis in Section 2.5.

### 2.7. Analysis of Health-Promoting Potential by In Vitro Methods

For the in vitro health-promoting activity analyses, two types of extracts were evaluated. The hydrophilic fraction corresponded to the methanolic extracts prepared for polyphenol determination (Section 2.3), whereas the hydrophobic fraction corresponded to the lipophilic extracts obtained for carotenoid, chlorophyll, tocopherol, and tocotrienol analyses (Section 2.5 and Section 2.6). All antioxidant and enzyme inhibitory assays were performed separately for the hydrophilic and hydrophobic fractions, allowing the contribution of both groups of bioactive compounds to be assessed.

#### 2.7.1. Antioxidant Activity

The antioxidant properties of the samples were evaluated using three methods: ABTS, FRAP (Ferric Reducing Antioxidant Power assay), and ORAC (Oxygen Radical Absorbance Capacity assay).

The ABTS method was performed according to Re et al. [12]. Briefly, 30 μL of appropriately diluted extract was mixed with 3 mL of ABTS•+ solution. Methanol and distilled water were used as controls. Absorbance was measured at 734 nm using a UV-2401 PC spectrophotometer (Shimadzu, Kyoto, Japan) 6 min after the addition of the ABTS radical cation.

The FRAP assay was based on the method presented by Benzie et al. [13]. Briefly, 1 mL of appropriately diluted extract was mixed with 3 mL of freshly prepared FRAP reagent. Methanol and distilled water were used as controls. After 10 min of reaction, absorbance was measured at 593 nm using a UV-2401 PC spectrophotometer (Shimadzu, Kyoto, Japan).

The ORAC assay was conducted following the spectrofluorimetric method described by Ou et al. [14]. Briefly, 0.25 mL of appropriately diluted extract was mixed with 1.5 mL of fluorescein solution and 0.25 mL of AAPH solution. Measurements were performed using an RF5301 PC spectrofluorometer (Shimadzu, Kyoto, Japan) at an excitation wavelength of 487 nm and an emission wavelength of 528 nm. The reaction mixtures were incubated at 37 °C, and fluorescence was recorded every 5 min after the addition of AAPH until complete fluorescence decay. Phosphate buffer was used as the blank. The results for all methods are expressed as mmol of Trolox/100 g fresh sample ± SD, based on three repetitions.

#### 2.7.2. Ability to Inhibit α-Amylase and α-Glucosidase

The analysis was conducted using the colorimetric method described previously by Nowicka et al. [7]. The study utilized a Synergy™ H1 microplate reader (BioTek, Winooski, VT, USA). For α-amylase inhibition, measurements were taken at a wavelength of 540 nm, while for α-glucosidase inhibition, measurements were taken at 405 nm. Acarbose was used as the positive control for both enzymes, with IC_50_ values of 0.35 and 0.20 mg/mL for α-amylase and α-glucosidase inhibition, respectively. The results are reported as IC_50_ ± SD (mg/mL).

IC_50_ values were determined using at least three concentrations of each sample, with each concentration analyzed in triplicate. The concentration ranges were individually adjusted for each sample and assay to obtain reliable dose–response curves and accurate IC_50_ determination. IC_50_ values were calculated from dose–response curves generated for each sample and assay. For all enzyme inhibitory assays, the control consisted of the corresponding reaction buffer used for sample dilution instead of the tested extract, representing 100% enzyme activity. Reagent blanks were prepared without enzyme, replacing it with the corresponding buffer, and were used to correct background absorbance.

#### 2.7.3. Ability to Inhibit Pancreatic Lipase

Pancreatic lipase inhibition was evaluated following the method described by Wojdyło et al. [15]. Absorbance measurements were taken at 400 nm using a Synergy™ H1 microplate reader (BioTek, Winooski, VT, USA). Orlistat was used as the positive control, with an IC_50_ value of 0.15 mg/mL. The results are expressed as IC_50_ ± SD (mg/mL). IC_50_ values were determined using at least three concentrations of each sample, with each concentration analyzed in triplicate.

#### 2.7.4. Ability to Inhibit Acetylcholinesterase and Butyrylcholinesterase

The ability of the samples to inhibit AChE and BuChE was assessed using the method described by Nowicka and Wojdyło [16]. For both cholinesterase inhibition assays, absorbance measurements were taken at a wavelength of 405 nm using a Synergy™ H1 microplate reader (BioTek, Winooski, VT, USA). The results are expressed as IC_50_ ± SD (mg/mL). IC_50_ values were determined using at least three concentrations of each sample, with each concentration analyzed in triplicate.

#### 2.7.5. Ability to Inhibit 15-Lipoxygenase

The inhibition of LOX-15 by the tested materials was evaluated using the method described by Nowicka et al. [7]. Absorbance measurements were taken at a wavelength of 560 nm using a Synergy™ H1 microplate reader (BioTek, Winooski, VT, USA). The results are expressed as IC_50_ ± SD (mg/mL). IC_50_ values were determined using at least three concentrations of each sample, with each concentration analyzed in triplicate.

### 2.8. Statistical Analysis

Statistical analysis was conducted using Statistica 13 (StatSoft, Kraków, Poland). Mean parameter values among the samples were compared by performing one-factor analysis of variance (ANOVA), followed by the Duncan test (*p* ≤ 0.05). Correlations between variables were analyzed using the Pearson test.

## 3. Results and Discussion

### 3.1. Polyphenolic Composition of Blackcurrant Matrices

In this study, the polyphenolic contents of five different groups—anthocyanins, phenolic acids, flavonols, flavan-3-ols, and polymeric procyanidins—were quantified in the analyzed plant materials (Table 1). This study demonstrated that the polyphenol contents in the analyzed materials differed statistically significantly (*p* ≤ 0.05). The highest amount of anthocyanins was found in fruits, followed by juice > seeds. Fruits (255 mg/100 g) contained 79% more of these compounds than juice (143 mg/100 g) and 11 times more than seeds (24 mg/100 g). The presence of anthocyanins was not detected in leaves and oil. The literature indicates that anthocyanins may have therapeutic effects on the circulatory, nervous, endocrine, digestive, sensory, urinary, and immune systems [1]. Blackcurrant anthocyanins exhibit beneficial effects against hypertension, diabetes, glaucoma, myopia, and hyperuricemia, as well as Parkinson’s and allergic diseases. Moreover, blackcurrant anthocyanins show activity against Alzheimer’s syndrome [17].

The research found that the dominant group of polyphenolic compounds varied depending on the plant material. Flavonols were the primary polyphenolic compounds in blackcurrant leaves (50% of the total polyphenol content), while procyanidin polymers were the dominant group in blackcurrant seeds (94%). In blackcurrant seed oil, the main fraction comprised flavan-3-ols (66%). In blackcurrant fruit and blackcurrant juice, anthocyanins were the dominant group (47% and 46%, respectively). Similar findings were reported by Gacnik et al. [18], who noted that flavonols were the dominant group of polyphenolic compounds in blackcurrant leaves, while anthocyanins were the primary compounds in their fruits [18].

Moreover, studies by other authors indicate that the concentrations of polyphenols from different group may vary across different varieties of berries within the same species. For example, Karaağaç and Sahan [19] reported that blackcurrant fruits of the “Rosenthal” variety contained 1586.76 mg anthocyanins/100 g DW, while the “Boskoop G.” variety contained 1777.73 mg/100 g DW.

The highest contents of phenolic acids, flavonols, and flavan-3-os were observed in the leaves. The concentration of phenolic acids in this plant material (108 mg/100 g) was 2 times higher than in seed oil (48 mg/100 g), and several times higher than in fruits, seeds, and juice (12, 13, and 17, respectively). The level of flavonols in leaves (331 mg/100 g) was 15, 16, and 31 times higher than in juice, fruits, and seeds, respectively, and their content of flavan-3-ols (106 mg/100 g) was 15%, 45%, and 75% higher than in oil, juice, and seeds, and 5 times higher than in fruits.

All of these compounds exhibit a wide range of health-promoting properties. Taking phenolic acids as an example, an animal study demonstrated that administering caffeic and chlorogenic acids (10 and 15 mg/kg/day) to rats with cyclosporine-induced hypertension resulted in reduced systolic blood pressure and heart rate, as well as inhibition of angiotensin-converting enzyme activity [20]. In turn, flavonols, especially derivatives of quercetin, kaempferol, and isorhamnetin, are among the flavonoids most frequently associated with health-promoting effects. They are known for their antioxidant, cardioprotective, anti-inflammatory, antibacterial, antiviral, and anticancer properties [21,22]. Meanwhile, in the context of flavan-3-ols, particularly catechin, epicatechin, and procyanidin derivatives, research by Crowe-White et al. [23] provides moderate evidence that consuming 400–600 mg/day of these compounds from food can help reduce the risk of cardiovascular disease and diabetes. These compounds can positively influence blood pressure (especially systolic), cholesterol levels, blood sugar levels, and enhance arterial flexibility [23].

In most of the analyzed plant matrices (except for seed oil), polymeric procyanidins were also identified. These compounds were the most abundant in blackcurrant seeds (1537 mg/100 g), where their amount was 6, 13, and 24 times higher than in fruits, leaves, and juice, respectively. Procyanidins are oligomeric or polymeric products formed during the final stage of the flavonoid biosynthesis pathway [24]. They can exhibit a range of health-promoting activities, including antioxidant, anticancer, anti-inflammatory, antiobesity, hypoglycemic, hypolipidemic, and blood pressure-lowering effects [6]. Moreover, procyanidins can selectively alter intestinal microbiota by inhibiting digestive enzymes or through antimicrobial activity. Due to their limited absorption in the upper gastrointestinal tract, a substantial proportion of procyanidins reaches the colon, where they are metabolized by the gut microbiota into low-molecular-weight phenolic metabolites. These metabolites may contribute to the biological activity of procyanidins and have been associated with antioxidant, anti-inflammatory, and cardioprotective effects. Furthermore, procyanidins may promote the growth of beneficial bacterial populations, thereby contributing to the maintenance of intestinal homeostasis and overall host health [25,26]. Both procyanidins and monomeric flavan-3-ols are particularly effective in inhibiting pancreatic α-amylase activity, though they also impact other digestive enzymes. However, their effects may vary depending on the matrix from which they are derived [24].

### 3.2. Carotenoids and Chlorophylls in Blackcurrant Matrices

Carotenoids and chlorophylls are natural, nontoxic dyes. Carotenoids are responsible for yellow, orange, and red colors, while chlorophylls provide green coloration. These compounds are used in the food industry to color food products [27]. In addition to their coloring properties, carotenoids function as antioxidants and may exhibit provitamin A activity. Lutein, a carotenoid, has been shown to counteract circulatory system diseases and cataracts, while the combined activity of lutein and lycopene, a second carotenoid, has been linked to protection against oxidative damage [27,28]. Chlorophylls also demonstrate antioxidant and antimutagenic effects [27]. The concentrations of carotenoids and chlorophylls in the analyzed materials are presented in Table 1.

This study revealed significant differences (*p* ≤ 0.05) in carotenoid content between plant materials. The amount of these compounds in seeds (319 mg/100 g) was 2, 10, 42, and 194 times higher than in leaves, fruits, seed oil, and juice, respectively. Moreover, in the case of leaves, the chlorophyll concentration (28 mg/100 g) was 5 times lower than the carotenoid level (147 mg/100 g). The remaining analyzed matrices did not contain chlorophyll. Gustinelli et al. [29] reported higher carotenoid content in blackcurrant seed oils extracted using three different methods, with values ranging from 13.2 to 38.0 mg/100 g, which exceeded the levels found in our study [29]. However, the content of bioactive compounds in oils, as well as their antioxidant properties, and oxidative stability depends on various factors, including seed preparation, the oil extraction method, the origin and cultivation conditions of the raw material, and its maturity [29,30].

### 3.3. Tocopherol and Tocotrienol Profiles of Blackcurrant Matrices

Eight compounds, including four tocopherols (α-, β-, γ-, and δ-tocopherol) and four tocotrienols (α-, β-, γ-, and δ-tocotrienol), exhibit vitamin E activity. Vitamin E is a fat-soluble antioxidant that must be obtained through the diet because the human body cannot synthesize it. Tocopherols and tocotrienols differ not only in structure but also in their mechanisms of action and reaction kinetics. Among these, D-α-tocopherol is considered the most bioavailable and is the predominant form of vitamin E. Interestingly, tocotrienols have been shown to possess more potent antioxidant and anti-inflammatory properties compared to tocopherols [31,32]. Tocotrienols can lower cholesterol levels, exhibit neuroprotective effects, and may have anticancer activity [31,32]. Moreover, unlike α-tocopherol, α-tocotrienol demonstrates neuroprotective effects even at nanomolar concentrations. Among these compounds, γ-tocotrienol and δ-tocotrienol are considered to have the strongest anticancer effects.

The tocopherol and tocotrienol concentrations are presented in Table 1. Additionally, in the study, the content of 13′-hydroxy-α-tocopherol, a degradation product of α-tocopherol, was also analyzed [33]. The results showed that plant material type significantly influenced the levels of individual tocopherols and tocotrienols (*p* ≤ 0.05). The content of α-tocopherol and the sum of β- and γ-tocopherol were the highest in blackcurrant seed oil (17 and 135 mg/kg, respectively), whereas the δ-tocopherol and α-tocotrienol were most abundant in leaves (12 and 4 mg/kg, respectively). The largest amounts of 13-hydroxy-α-tocopherol, the sum of β- and γ-tocotrienol, and δ-tocotrienol were recorded in seeds (4, 2 and 3 mg/kg).

Gustinelli et al. [29] reported that blackcurrant oils extracted using supercritical fluid extraction (SFE) at 50 and 80 °C, as well as hexane extraction, exhibited higher levels of α-tocopherol (307–1221 mg/kg), δ-tocopherol (125–160 mg/kg), and γ-tocotrienol (1–24 mg/kg) compared to the results of this study. Their oils also contained higher concentrations of γ-tocopherol (697–1027 mg/kg) than the sum of β- and γ-tocopherol recorded in the present study. Furthermore, Gustinelli et al. [29] identified 3 mg/kg of α-tocotrienol in blackcurrant oils extracted by SFE, while this compound was absent in hexane-extracted blackcurrant oil and the blackcurrant seed oil analyzed in our study.

### 3.4. Potential Bioaccessibility and Potential Intestinal Availability Estimated by Dialysis of Polyphenolic Compounds

This section discusses the amounts of potentially bioaccessible polyphenolic compounds in blackcurrant fruits, juice, and leaves and their potential intestinal availability estimated by dialysis. The results of the in vitro study are presented in Table 2.

A comparison of the tested materials revealed statistically significant differences (*p* ≤ 0.05) in the content of potentially bioaccessible polyphenols. For anthocyanins and procyanidin polymers, the highest levels were observed in blackcurrant fruits. The fruits contained three times more bioaccessible anthocyanidins than the juice and ten times more bioaccessible procyanidin polymers than the leaves. In contrast, blackcurrant leaves were the richest source of bioaccessible phenolic acids, flavonols, and flavan-3-ols. Their concentrations were 30% (flavan-3-ols) to 28-fold (flavonols) higher than in the juice and 2-fold (flavan-3-ols) to 133-fold (phenolic acids) higher than in the fruits. Overall, the total bioaccessible polyphenol content in leaves was three times greater than in the other matrices. Statistically significant differences (*p* ≤ 0.05) were also observed in the levels of potential intestinal availability of anthocyanins, phenolic acids, flavonols, flavan-3-ols, and total polyphenols depending on the plant matrix, estimated by dialysis. With the exception of anthocyanins and flavonols, blackcurrant leaves showed the highest levels of compounds in the dialyzable fraction, indicating the greatest potential intestinal availability estimated by dialysis, followed by juice and fruits. Although leaves also had the highest levels of flavonols in the dialyzable fraction, the amounts in fruits and juice did not differ significantly. In contrast, fruits were the richest source of anthocyanins in the dialyzable fraction, followed by juice and leaves.

The results indicate that an equivalent dose of blackcurrant leaves can provide 125-, 25-, and 3-fold higher amounts of phenolic acids, flavonols, and total polyphenols in the dialyzable fraction, respectively, compared with fruits, and 17-, 28-, and 2-fold higher amounts than juice. Leaves also contained 88% and 12% higher levels of flavan-3-ols in the dialyzable fraction than fruits and juice, respectively. The dialyzable fraction accounted for 70% (leaves) to 80% (juice) of the total polyphenols reaching the intestinal stage of the in vitro digestion model.

The results of this in vitro study suggest that 63% to 96% of anthocyanins, phenolic acids, flavonols, and flavan-3-ols from blackcurrant fruits, juice, and leaves that reach the intestine may be present in the dialyzable fraction, indicating their potential intestinal availability estimated by dialysis. Estimating the dialyzable fraction of polymeric procyanidins remains challenging due to their very low levels after simulated digestion. Other authors have highlighted the incomplete understanding of proanthocyanidin transformations during digestion: in vitro models indicate hydrolysis and the formation of flavan-3-ols, whereas in vivo studies do not confirm depolymerization [34].

Previous studies also reported that extracts from blackcurrant, raspberry, and chokeberry leaves are dominated by small phenolic compounds (gallic, ellagic, and chlorogenic acids, respectively) which, due to their size, may exhibit good absorption [4]. However, this hypothesis was not experimentally verified. In our study, the amounts of phenolic acids present in the dialyzable fraction of blackcurrant juice and leaves, as well as flavan-3-ols from all tested samples, exceeded the levels initially measured in the raw materials. This finding suggests that simulated digestion induced chemical reactions leading to the degradation of complex polyphenols and the release of simpler compounds such as flavan-3-ols and phenolic acids. In other cases, the proportion of compounds recovered in the dialyzable fraction was lower than their initial content in the raw materials, ranging from <0.01% (polymeric procyanidins) to 57% (flavonols in fruits).

Because the present digestion model did not include the gut microbiota, the study does not provide information on the subsequent fate of polyphenolic compounds not identified in the dialyzable fraction, including their potential microbial transformation or excretion. However, other studies show that undigested flavonoids reaching the large intestine can be converted into phenolic acids through microbiota-mediated processes [22,35,36,37]. Moreover, polyphenols themselves can modulate the growth, composition, and metabolic activity of the gut microbiota [20,34]. Therefore, extending future research to explore these interactions would provide valuable insight.

### 3.5. Antioxidant Capacity of Hydrophilic and Hydrophobic Fractions of Blackcurrant Matrices

Scientists often use various methods to determine the health-promoting potential of food by assessing its antioxidant capacity in vitro. Antioxidants in different products prevent oxidative stress by neutralizing free oxygen radicals, which, if accumulated in the body, can contribute to the development of numerous diseases, including diabetes, atherosclerosis, Alzheimer’s disease, Parkinson’s disease, and cancer [4,21,28]. Oxidative stress is also detrimental to food quality, as it can lead to a deterioration in properties and a shorter shelf life [21].

To make a more precise assessment of raw materials or products, it is recommended to use multiple analyses based on different mechanisms of action, reaction times, and media [20]. In this study, the ABTS, FRAP, and ORAC methods were used to evaluate antioxidant capacity. The results for hydrophilic compounds are presented in Table 3, and those for hydrophobic substances in Table 4.

Statistical analysis revealed that the antioxidant properties of both hydrophilic and hydrophobic compounds, as analyzed by all three methods, differed significantly (*p* ≤ 0.05) between the plant materials studied. The ABTS and FRAP methods showed that the effects of hydrophilic compounds of blackcurrant seeds were higher than those of leaves (2 and 3 times, respectively), juice (3 and 15 times, respectively), and fruits (14 and 13 times, respectively), while the ORAC method showed that the activities exhibited by hydrophilic compounds of seeds and juice constituted a homogeneous statistical group with the highest effectiveness. According to the ORAC method, the antioxidant capacity of these compounds from leaves was 33% and 27% lower, respectively, than those of seeds and juice. However, fruit compounds were 8 and 7 times less effective, respectively.

In the case of hydrophobic compounds, blackcurrant leaves exhibited the greatest ABTS capacity, while the FRAP method identified that the antioxidant effect of oil was the highest. The ORAC method showed that seeds and seed oil belonged to the homogeneous group with the highest antioxidant capacity. However, all values obtained in the study for hydrophobic compounds were low, <0.85 mmol Trolox/100 g. Hence, hydrophilic compounds contained in fruits, seeds, and leaves showed significantly greater antioxidant capacity than their hydrophobic compounds.

The observed differences in antioxidant capacity were largely consistent with the phytochemical profiles of the analyzed materials (Table 1). The strongest capacity of hydrophilic fractions from seeds may be explained by their exceptionally high content of polymeric procyanidins, which constituted the dominant group of polyphenols in this matrix. In contrast, the high antioxidant capacity of leaf extracts likely reflects the combined contribution of flavonols, phenolic acids, and flavan-3-ols, all present at higher concentrations than in the remaining materials. These findings suggest that the antioxidant potential of blackcurrant matrices depends not only on the total concentration of bioactive compounds but also on their qualitative composition and relative proportions. Moreover, the markedly lower capacity of hydrophobic fractions indicates that polyphenols may play a more important role in determining antioxidant capacity than carotenoids, chlorophylls, tocopherols, and tocotrienols, despite the recognized antioxidant properties of these lipophilic compounds.

Previously, the antioxidant properties of blackcurrant fruits, leaf extracts, seed oil, or products have also been studied using various methods and documented in the literature [4,19,29,38,39]. For example, Brzezowska et al. [39] showed that blackcurrant juice powders produced via freeze-drying had higher antioxidant capacity, measured by TEAC ABTS and FRAP (23.21 and 19.25 mmol Trolox/100 g dm, respectively), than those made from blackcurrant fruits (17.59 and 15.32 mmol Trolox/100 g dm, respectively).

In this study, we compared the antioxidant capacity of hydrophilic and hydrophobic compounds extracted from fruits, juice, seeds, and leaves using two different methods. For hydrophilic compounds, very strong correlations were observed between the antioxidant effects measured by the FRAP method and the contents of procyanidin polymers (*r* = 0.93), δ-tocotrienol (*r* = 0.94), the sum of β- and γ-tocopherol (*r* = 0.93), and carotenoids (*r* = 0.99). Strong correlations were also found between the results of the ABTS, FRAP, and ORAC tests and the anthocyanin content in the samples (*r* = −0.79, *r* = −0.70, and *r* = −0.70, respectively). While anthocyanins are recognized as antioxidants, the correlation value in this case was negative. It is important to note that correlation does not imply causation. Moreover, it is the entire matrix of compounds, rather than individual substances, that plays a critical role in shaping the health-promoting properties of food. In this study, the negative correlation likely reflects a higher total presence of antioxidant compounds in matrices with low or no anthocyanin content.

Additionally, strong correlations were identified between the antioxidant effects of hydrophilic compounds (ABTS) and the concentration of procyanidin polymers (*r* = 0.83), δ-tocotrienol (*r* = 0.88), the sum of β- and γ-tocopherol (*r* = 0.83), and carotenoids (*r* = 0.89). The FRAP activities strongly correlated with the concentration of 13-hydroxy-α-tocopherol (*r* = 0.80).

The relationship between the activities of hydrophobic compounds in fruits, seeds, oil, and leaves, as analyzed by different methods, showed a different pattern. Very strong correlations were observed between the ABTS capacity of hydrophobic compounds from the previously cited matrices and the concentrations of phenolic acids and flavonols (*r* = 0.96 and *r* = 0.96, respectively), as well as FRAP capacity and anthocyanin level (*r* = −0.93). The results of the ABTS and FRAP analyses were strongly correlated with the content of flavan-3-ols (*r* = 0.73 and *r* = 0.78, respectively). Additionally, the antioxidant capacity measured by ABTS strongly depended on the concentrations of δ-tocopherol (*r* = 0.90) and α-tocotrienol (*r* = 0.87, respectively). A strong correlation was also noted between anthocyanin concentration and the ORAC capacity of hydrophobic compounds (*r* = −0.75).

### 3.6. Ability to Inhibit α-Amylase and α-Glucosidase

α-Amylase and α-glucosidase are digestive enzymes responsible for breaking down carbohydrates into monosaccharides, which are absorbed during digestion and lead to an increase in blood glucose levels. When excess glucose accumulates in the blood instead of being converted into energy, it can contribute to the development of type 2 diabetes. Risk factors for this condition include smoking, obesity, lack of physical activity, and poor nutrition, while its prevention often involves inhibiting the activity of these digestive enzymes [5,40,41]. Studies have shown that polyphenols can inhibit the catalytic activity of these enzymes by binding to their active sites via hydrogen bonding with amino acid residues, thereby forming complexes [41].

The ability of the substances in the studied materials to inhibit α-amylase and α-glucosidase is presented in Table 3 and Table 4 (activities of hydrophilic and hydrophobic compounds, respectively). The study demonstrated that the enzyme inhibitions by the hydrophilic and hydrophobic compounds varied significantly (*p* ≤ 0.05) depending on the plant material type. The activities of hydrophilic compounds against α-amylase from juice and seeds were the highest and did not vary significantly, reaching IC_50_ values < 0.01 mg/mL. It is important to highlight that the IC_50_ value for acarbose, an antidiabetic drug, in terms of α-amylase inhibition in our study was 0.35 mg/mL. This indicates a promising in vitro enzyme inhibitory potential of these plant materials. However, further cellular, in vivo, and clinical studies are required to determine whether these effects translate into physiologically relevant outcomes.

Moreover, no significant differences were found between the abilities of hydrophilic compounds from fruits (IC_50_: 0.82 mg/mL) and seeds (IC_50_: 0.94 mg/mL) to inhibit α-glucosidase, which were the most effective. However, these values indicate a lower effect than acarbose (IC_50_: 0.20 mg/mL). In this study, among the hydrophobic compounds, only the substances from blackcurrant fruit showed relatively promising activity against α-amylase. Their IC_50_ value was 0.80 mg/mL. However, this value was still higher than that of acarbose and therefore does not indicate a strong antidiabetic potential for these compounds.

The effective action of blackcurrant and rowanberry extracts in inhibiting α-glucosidase has been highlighted by other authors, who reported IC_50_ values of 0.02 mg GAE/mL and 0.03 mg GAE/mL, respectively. In their studies, the inhibition efficiency of acarbose was lower, with an IC_50_ of approximately 0.04 mg/mL. In addition, both extracts, when used separately, were found to enhance the activity of acarbose. It suggests the potential to use them either as a replacement for the drug or in combination with a lower dose, which could help minimize the risk of potential side effects [40]. However, further research would be necessary to evidence the possibility of introducing such alternatives.

Other studies have shown that powders made from blackcurrant juice, pomace, and fruits had inhibitory effects on α-amylase with IC_50_ values of <0.05, <0.05, and 5.54 mg/mL, respectively, and on α-glucosidase with IC_50_ values of 5.75, 8.99, and 16.66 mg/mL, respectively. Additionally, these studies showed that all phenolic groups contributed to the inhibitory effect on α-glucosidase [39]. In our study, hydrophilic compounds from blackcurrant juice also exhibited greater α-amylase inhibition than those from blackcurrant fruits; however, for α-glucosidase inhibition, the trend was reversed.

Correlation studies using the Pearson test revealed strong correlations between the ability of hydrophilic compounds to inhibit α-amylase and the contents of anthocyanins and flavan-3-ols (*r* = 0.76 and *r* = −0.72, respectively). Very strong positive correlations were observed between the inhibition of α-glucosidase by hydrophilic compounds and the levels of phenolic acids and flavonols (*r* = 0.92 and *r* = 0.93, respectively). Additionally, the inhibition of α-glucosidase by these substances strongly depended on the concentrations of flavan-3-ols (*r* = 0.88), 13-hydroxy-α-tocopherol (*r* = −0.74), α-tocotrienol (*r* = 0.82), δ-tocopherol (*r* = 0.88), and α-tocopherol (*r* = −0.84) and the sum of β- and γ-tocotrienol (*r* = −0.81).

These findings suggest that phenolic acids, flavonols, and flavan-3-ols may be among the major contributors to the observed α-glucosidase inhibitory activity. In particular, blackcurrant leaves, which were characterized by the highest concentrations of phenolic acids and flavonols (Table 1), may represent a valuable source of compounds involved in carbohydrate metabolism regulation. Anthocyanins and flavan-3-ols also appear to contribute to α-amylase inhibition, which is consistent with previous reports describing their interactions with digestive enzymes through hydrogen bonding and hydrophobic interactions.

For hydrophobic compounds from fruits, seeds, oil, and leaves, very strong correlation was observed between their α-amylase inhibition activity and the sum of β- and γ-tocopherol (*r* = 0.92). Additionally, very strong negative correlations were found between their α-glucosidase inhibition ability and the concentration of phenolic acids (*r* = −0.92), flavan-3-ols (*r* = −1.00), and α-tocotrienol (*r* = −0.94), and strong correlations were observed between the inhibition of this enzyme and the amounts of δ-tocopherol (*r* = −0.83) and α-tocopherol (*r* = 0.80).

### 3.7. Ability to Inhibit Pancreatic Lipase

Pancreatic lipase is an enzyme produced by pancreatic acinar cells. It hydrolyzes dietary fats into monoacylglycerols and free fatty acids in the intestinal lumen. Together with gastric lipase, it facilitates the absorption of triacylglycerols by enterocytes and the assimilation of fat. Lipase-inhibiting agents reduce or prevent the absorption of energy from fats, making the inhibition of lipase activity a potential strategy for preventing overweight and obesity, particularly in diabetics [5,42].

Tetrahydrolipstatin, a synthetic analog of lipstatin derived from *Streptomyces toxytricini*, is a clinically used lipase inhibitor for treating obesity due to its effective pancreatic lipase-inhibiting activity. However, lipase inhibition may be undesirable in people with conditions such as cystic fibrosis, who require supplementation with pancreatic enzymes (including lipase, amylase, and protease) to manage exocrine pancreatic insufficiency. Similarly, it may not be suitable for individuals with celiac disease or Crohn’s disease who rely on pancreatic enzyme supplementation [42].

The results of pancreatic lipase inhibition activity by hydrophilic and hydrophobic compounds from the tested plant materials are presented in Table 3 and Table 4, respectively. Studies examining the ability of hydrophilic compounds to inhibit pancreatic lipase showed many statistically significant differences between plant materials (*p* ≤ 0.05). Substances from the juice were most effective and capable of inhibiting 50% of the enzyme’s activity (IC_50_) at a dose of 0.01 mg/mL. Moreover, they exhibited greater efficacy than Orlistat (Tetrahydrolipstatin), whose IC_50_ was 0.15 mg/mL. This finding suggests that the in vitro activity of this juice offers potential for the development of an effective antiobesity drug based on plant components. In contrast, the action of hydrophobic substances did not differ significantly and was considered ineffective.

In our research, the Pearson test revealed a very strong correlation between the ability of hydrophilic compounds in fruits, juice, seeds, and leaves to inhibit lipase and the concentrations of α-tocotrienol (*r* = 0.98) and δ-tocopherol (*r* = 0.93). Additionally, strong correlations were observed between their activity and the concentrations of anthocyanins (*r* = −0.85), phenolic acids (*r* = 0.87), flavonols (*r* = 0.85), and flavan-3-ols (*r* = 0.74).

The observed correlations indicate that both polyphenolic compounds and selected vitamin E homologues may contribute to pancreatic lipase inhibition. Phenolic acids, flavonols, and flavan-3-ols have previously been reported to interact with lipase and reduce its catalytic activity, supporting their potential role in the anti-obesity effects observed in the present study.

### 3.8. Ability to Inhibit Acetylcholinesterase and Butyrylcholinesterase

Studies have shown that the brains of individuals with Alzheimer’s and Parkinson’s diseases are characterized by low levels of acetylcholine. Therefore, the use of natural raw materials capable of inhibiting AChE could potentially facilitate the development of new, effective drugs for these neurodegenerative diseases. For instance, alkaloids and terpenoids are recognized as effective cholinesterase inhibitors [5]. However, reports suggest that the efficacy of AChE inhibitors, which constituted three of the four Alzheimer’s disease drugs approved by the Food and Drug Administration (FDA) as of 2022, is not entirely satisfactory. Additionally, it is known that individuals with Alzheimer’s disease often exhibit elevated levels of BuChE in the brain. As such, BuChE inhibition may also play a role in preventing disease progression [43].

The results regarding the ability of the samples to inhibit the activity of AChE and BuChE are presented in Table 2 and Table 3 for hydrophilic and hydrophobic compounds, respectively. This study found that plant material type was a significant factor (*p* ≤ 0.05) influencing the ability of hydrophilic and hydrophobic compounds to inhibit AChE and BuChE. Juice was the significantly most effective in inhibiting both cholinesterases and its IC_50_ values were ≤0.11 mg/mL. The activity of blackcurrant juice against BuChE was particularly remarkable, as it inhibited 50% of BuChE activity at a dose of just 0.03 mg/mL.

The lowest IC50 values among hydrophobic substances were recorded in the oil; however, they do not appear promising for developing products targeting AChE and BuChE inhibition, as they were >65.00 mg/mL. Studies by other authors suggest that the stage of maturity of plant materials may influence their ability to inhibit AChE and BuChE. Hwang et al. [44] demonstrated that among unripe and ripe Korean blueberries (“Nelson,” “Duke,” “Bluejay,” “Toro,” and “Elliot”), the unripe raw materials exhibited significantly higher AChE and BuChE inhibition efficiency. However, a broader discussion of literature data regarding the ability of berry fruits, their products, or other morphological parts of their bushes to inhibit AChE and BuChE is challenging due to the limited availability of such studies. Information about the existence of only a few reports on this subject, which concerns berry fruits, was also reported by the previously mentioned authors [44].

In our study, the Pearson test revealed no very strong or strong correlations between the ability of hydrophilic compounds from fruits, juice, seeds, and leaves to inhibit AChE or BuChE and the content of any analyzed ingredient. For hydrophobic compounds from fruits, seeds, oil, and leaves, the abilities to inhibit AChE and BuChE were very strongly correlated with the sum of β- and γ-tocopherol (*r* = −0.99 for both enzymes) and strongly correlated with the concentration of α-tocopherol (*r* = −0.82 for both enzymes). The strong associations observed for tocopherols suggest that lipophilic vitamin E compounds may contribute to cholinesterase inhibition. Although the mechanisms responsible for this activity remain less understood than for polyphenols, several studies have indicated that tocopherol-rich extracts may exert neuroprotective effects through both antioxidant and enzyme-modulating activities.

### 3.9. Ability to Inhibit LOX-15

In vitro studies assessing the anti-inflammatory potential of food often involve determining its ability to inhibit LOX or COX enzymes. These enzymes play a role in lipid metabolism and are responsible for oxidizing linoleic and arachidonic acids, leading to the formation of metabolites such as cis- and trans-conjugated hydroperoxides and prostaglandins. LOX and COX are associated with various inflammatory diseases, including allergies, bronchial asthma, atherosclerosis, osteoporosis, and cancer [15]. This study focused on the ability of hydrophilic and hydrophobic substances from the tested plant materials to inhibit LOX-15, with results presented in Table 3 and Table 4, respectively.

The study demonstrated that the LOX-15 inhibitions by the hydrophilic and hydrophobic substances varied significantly (*p* ≤ 0.05) depending on the plant matrix. For hydrophilic substances, the highest LOX-15-inhibiting effect among hydrophilic substances was found in the blackcurrant juice. Regarding the ability of hydrophobic compounds to inhibit this enzyme, the lowest IC50 values were recorded in the group consisting of blackcurrant oil and leaves. Notably, only blackcurrant juice appears promising, as it exhibited 50% inhibition of LOX-15 (IC_50_) at concentrations of 0.02 mg/mL.

Correlation analysis using the Pearson test revealed strong correlations between the ability of hydrophilic compounds in the samples to inhibit LOX-15 and the content of anthocyanins (*r* = 0.80) and flavan-3-ols (*r* = −0.87). For hydrophobic compounds from fruits, seeds, oil, and leaves, strong correlations were observed between their ability to inhibit LOX-15 and the concentrations of phenolic acids (*r* = −0.71), flavan-3-ols (*r* = −0.81), 13-hydroxy-α-tocopherol (*r* = 0.78), and δ-tocopherol (*r* = −0.81) and the sum of β- and γ-tocotrienol (*r* = 0.90). The correlations obtained for anthocyanins, flavan-3-ols, phenolic acids, and vitamin E derivatives suggest that these compounds may contribute to the anti-inflammatory potential of blackcurrant matrices. In particular, flavan-3-ols and anthocyanins have been widely reported as modulators of lipoxygenase activity and inflammatory signaling pathways [1,5,7].

## 4. Conclusions

The conducted studies demonstrate that blackcurrant fruits, juice, seeds, oil, and leaves exhibit distinct chemical compositions and broad in vitro health-promoting potentials. Among all examined plant materials, blackcurrant fruits were characterized by the highest concentrations of anthocyanins. Blackcurrant seeds contained the greatest amounts of procyanidin polymers, carotenoids, 13-hydroxy-α-tocopherol, δ-tocotrienol, and the sum of β- and γ-tocotrienols, while blackcurrant seed oil displayed the highest levels of α-tocopherol and total β- and γ-tocopherols. In turn, blackcurrant leaves proved to be the richest source of phenolic acids, flavonols, flavan-3-ols, δ-tocopherol, and α-tocotrienol.

With respect to the potential intestinal availability of polyphenols estimated by dialysis, fruits contained the greatest amounts of anthocyanins in the dialyzable fraction, whereas leaves offered the highest levels of phenolic acids, flavonols, flavan-3-ols, and total polyphenols. The antioxidant analyses revealed that the hydrophilic fraction of blackcurrant seeds exhibited the strongest antioxidant activity across all three applied analytical methods. The analyzed blackcurrant matrices also demonstrated promising in vitro antioxidant, antidiabetic, anti-obesity, anti-inflammatory, and neuroprotective potential. Although these findings highlight the possible utility of blackcurrant-derived materials in the development of plant-based formulations targeting diabetes and obesity, the results should be interpreted within the limitations of in vitro assays and comprehensive in vivo and clinical studies are necessary to determine whether the observed in vitro activities translate into physiologically meaningful effects.

Overall, the present studies confirm that not only fruits and juice but also the seeds and leaves of blackcurrant constitute valuable sources of bioactive compounds with promising in vitro biological activities. When considering their application in food processing or the development of health-promoting products, it is essential to account for possible interactions between these compounds and other ingredients, which may modify their bioactivity and potential intestinal availability. Nevertheless, the collected findings clearly indicate that blackcurrant-derived plant materials warrant further intensive research aimed at exploring their technological, nutritional, and pharmaceutical potential.

## Figures and Tables

**Table 1:** Bioactive compound contents in selected morphological parts and products of blackcurrants (mg/100 g or mg/kg).

Compound	Fruits	Juice	Seeds	Seed oil	Leaves
polyphenols (mg/100 g)					
anthocyanins	255.11 ± 3.16 ^a^	142.79 ± 2.74 ^b^	23.57 ± 0.35 ^c^	0.00 ± 0.00 ^d^	0.00 ± 0.00 ^d^
phenolic acids	9.00 ± 0.07 ^c^	6.43 ± 0.18 ^d^	8.16 ± 0.10 ^c^	47.65 ± 1.32 ^b^	108.27 ± 1.34 ^a^
flavonols	20.79 ± 0.47 ^b^	22.32 ± 0.33 ^b^	10.57 ± 0.29 ^c^	0.00 ± 0.00 ^d^	330.87 ± 4.90 ^a^
flavan-3-ols (monomeric and dimeric)	20.02 ± 0.56 ^e^	72.95 ± 0.58 ^c^	60.43 ± 1.35 ^d^	91.66 ± 2.05 ^b^	105.78 ± 2.03 ^a^
procyanidin polymers	236.57 ± 6.62 ^b^	64.37 ± 1.29 ^d^	1537.37 ± 41.51 ^a^	nd.	120.10 ± 1.20 ^c^
carotenoids and chlorophylls (mg/100 g)					
carotenoids	31.01 ± 0.56 ^c^	1.64 ± 0.01 ^d^	319.12 ± 7.82 ^a^	7.67 ± 0.19 ^d^	147.45 ± 0.41 ^b^
chlorophylls	nd.	nd.	nd.	nd.	27.75 ± 0.50 ^a^
tocopherols (mg/kg)					
α-tocopherol	7.86 ± 0.22 ^c^	0.35 ± 0.01 ^d^	9.14 ± 0.05 ^b^	17.09 ± 0.27 ^a^	0.32 ± 0.01 ^d^
β- and γ-tocopherol	2.17 ± 0.01 ^c^	0.24 ± 0.00 ^d^	18.66 ± 0.37 ^b^	134.61 ± 1.92 ^a^	0.61 ± 0.01 ^cd^
δ-tocopherol	0.33 ± 0.00 ^d^	0.01 ± 0.00 ^e^	1.68 ± 0.02 ^c^	7.03 ± 0.08 ^b^	11.58 ± 0.18 ^a^
13-hydroxy-α-tocopherol	1.39 ± 0.03 ^b^	0.48 ± 0.01 ^c^	3.74 ± 0.05 ^a^	0.00 ± 0.00 ^d^	0.00 ± 0.00 ^d^
tocotrienols (mg/kg)					
α-tocotrienol	0.59 ± 0.01 ^c^	0.39 ± 0.01 ^d^	1.60 ± 0.04 ^b^	0.00 ± 0.00 ^e^	4.33 ± 0.06 ^a^
β- and γ-tocotrienol	1.08 ± 0.02 ^b^	0.00 ± 0.00 ^c^	1.59 ± 0.03 ^a^	0.00 ± 0.00 ^c^	0.00 ± 0.00 ^c^
δ-tocotrienol	0.00 ± 0.00 ^b^	0.00 ± 0.00 ^b^	2.33 ± 0.05 ^a^	0.00 ± 0.00 ^b^	0.00 ± 0.00 ^b^

Results are presented as the mean with standard deviation (n = 3). Different letters within a row indicate statistically significant differences among samples according to one-way ANOVA followed by Duncan’s multiple range test (*p* ≤ 0.05); nd.—not detected.

**Table 2:** Amounts of potentially bioaccessible and dialyzable polyphenol fraction in the selected morphological parts and products of blackcurrants (mg/100 g).

Compound		Fruits	Juice	Leaves
**potentially bioaccessible polyphenols content (mg/100 g)**	anthocyanins	181.49 ± 0.91 ^a^	60.49 ± 0.60 ^b^	0.00 ± 0.00 ^c^
	phenolic acids	1.33 ± 0.01 ^c^	8.09 ± 0.13 ^b^	177.08 ± 0.89 ^a^
	flavonols	18.89 ± 0.09 ^b^	15.77 ± 0.25 ^b^	434.10 ± 4.34 ^a^
	flavan-3-ols (monomeric and dimeric)	150.86 ± 0.75 ^c^	242.83 ± 2.43 ^b^	324.15 ± 7.13 ^a^
	procyanidin polymers	0.10 ± 0.00 ^a^	0.00 ± 0.00 ^c^	0.01 ± 0.00 ^b^
	Ʃ polyphenolic compounds	352.68 ± 1.76 ^b^	327.19 ± 3.41 ^c^	935.34 ± 12.36 ^a^
**potential intestinal availability estimated by dialysis (mg/100 g)**	anthocyanins	122.80 ± 0.61 ^a^	43.52 ± 0.44 ^b^	<0.01 ^c^
	phenolic acids	1.08 ± 0.01 ^c^	7.78 ± 0.12 ^b^	134.96 ± 0.67 ^a^
	flavonols	11.90 ± 0.06 ^b^	10.50 ± 0.17 ^b^	291.96 ± 2.92 ^a^
	flavan-3-ols (monomeric and dimeric)	119.36 ± 0.60 ^c^	199.56 ± 2.00 ^b^	224.40 ± 4.94 ^a^
	procyanidin polymers	<0.01 ^a^	<0.01 ^a^	<0.01 ^a^
	Ʃ polyphenolic compounds	255.14 ± 1.28 ^b^	261.37 ± 2.72 ^b^	651.32 ± 8.53 ^a^

Results are presented as the mean with standard deviation (n = 3). Letters indicate homogeneous groups determined by one-factor analysis of variance (ANOVA) using the Duncan test (*p* ≤ 0.05).

**Table 3:** In vitro health-promoting properties of hydrophilic compounds isolated from selected morphological parts and products of blackcurrants: antioxidant activities (mmol Trolox/100 g) and abilities to inhibit α-amylase, α-glucosidase, pancreatic lipase, AChE, BuChE, and LOX-15 (IC_50_ as mg/mL).

Parameter	Fruits	Juice	Seeds	Leaves
**antioxidant capacity:** ABTS (mmol Trolox/100 g)	0.98 ± 0.03 ^c^	5.30 ± 0.06 ^b^	13.32 ± 1.26 ^a^	6.51 ± 0.30 ^b^
FRAP (mmol Trolox/100 g)	0.82 ± 0.02 ^c^	0.75 ± 0.01 ^c^	10.88 ± 0.09 ^a^	4.15 ± 0.15 ^b^
ORAC (mmol Trolox/100 g)	1.44 ± 0.05 ^c^	10.52 ± 0.70 ^a^	11.41 ± 1.46 ^a^	7.69 ± 0.74 ^b^
**ability to inhibit:** α-amylase (mg/mL)	12.24 ± 0.00 ^c^	<0.01 ^a^	<0.01 ^a^	2.49 ± 0.10 ^b^
α-glucosidase (mg/mL)	0.82 ± 0.24 ^a^	6.64 ± 1.09 ^b^	0.94 ± 0.02 ^a^	16.09 ± 0.00 ^c^
pancreatic lipase (mg/mL)	1.81 ± 0.01 ^b^	0.01 ± 0.00 ^a^	24.15 ± 0.94 ^c^	47.77 ± 0.27 ^d^
AChE (mg/mL)	>200.00 ^c^	0.11 ± 0.00 ^a^	nd.	127.71 ± 1.94 ^b^
BuChE (mg/mL)	>200.00 ^c^	0.03 ± 0.00 ^a^	nd.	197.93 ± 0.87 ^b^
LOX-15 (mg/mL)	168.32 ± 0.44 ^d^	0.02 ± 0.01 ^a^	24.52 ± 0.00 ^c^	7.10 ± 1.21 ^b^

Results are presented as mean values ± SD. Antioxidant capacity assays were performed in triplicate (n = 3). IC50 values were determined using at least three concentrations, each analyzed in triplicate. Different letters indicate statistically significant differences among samples according to one-way ANOVA followed by Duncan’s multiple range test (*p* ≤ 0.05); nd.—not detected.

**Table 4:** In vitro health-promoting properties of hydrophobic compounds isolated from selected morphological parts and products of blackcurrants: antioxidant activities (mmol Trolox/100 g) and abilities to inhibit α-amylase, α-glucosidase, pancreatic lipase, AChE, BuChE, and LOX-15 (IC_50_ as mg/mL).

Parameter	Fruits	Seeds	Oil	Leaves
**Antioxidant capacity:** ABTS (mmol Trolox/100 g)	0.01 ± 0.00 ^b^	0.01 ± 0.00 ^b^	0.02 ± 0.01 ^b^	0.08 ± 0.01 ^a^
FRAP (mmol Trolox/100 g)	0.05 ± 0.00 ^d^	0.65 ± 0.03 ^b^	0.82 ± 0.04 ^a^	0.55 ± 0.07 ^c^
ORAC (mmol Trolox/100 g)	0.05 ± 0.01 ^c^	0.29 ± 0.06 ^a^	0.27 ± 0.04 ^a^	0.14 ± 0.01 ^b^
**ability to inhibit:** α-amylase (mg/mL)	0.80 ± 0.00 ^a^	37.74 ± 30.77 ^b^	168.52 ± 1.75 ^c^	58.63 ± 0.46 ^b^
α-glucosidase (mg/mL)	>200.00 ^a^	>200.00 ^a^	>200.00 ^a^	nd.
pancreatic lipase (mg/mL)	>200.00 ^a^	>200.00 ^a^	>200.00 ^a^	>200.00 ^a^
AChE (mg/mL)	>200.00 ^b^	>200.00 ^b^	65.75 ± 0.00 ^a^	>200.00 ^b^
BuChE (mg/mL)	>200.00 ^b^	>200.00 ^b^	109.01 ± 0.00 ^a^	>200.00 ^b^
LOX-15 (mg/mL)	>200.00 ^b^	>200.00 ^b^	109.41 ± 24.67 ^a^	129.41 ± 25.21 ^a^

Results are presented as mean values ± SD. Antioxidant capacity assays were performed in triplicate (n = 3). IC50 values were determined using at least three concentrations, each analyzed in triplicate. Different letters indicate statistically significant differences among samples according to one-way ANOVA followed by Duncan’s multiple range test (*p* ≤ 0.05); nd.—not detected.

## Data Availability

The original contributions presented in this study are included in the article. Further inquiries can be directed to the corresponding author.
